# Changes of healthy brain tissue after salvage radiotherapy of glioblastoma

**DOI:** 10.1093/noajnl/vdab139

**Published:** 2021-09-20

**Authors:** Leandra de la Cruz, Xiaoran Chen, Ender Konugoglu, I Frank Ciernik

**Affiliations:** 1 Medical School, University of Zurich (MeF), Zurich, Switzerland; 2 Biomedical Image Computing, Department of Information Technology and Electrical Engineering, Federal Institute of Technology (ETH-Z), Zürich, Switzerland; 3 Department of Radiotherapy and Radiation Oncology, Dessau City Hospital, Dessau, Germany; 4 Center of Oncology, Dessau City Hospital, Dessau, Germany

**Keywords:** artificial intelligence, deep learning, GBM, glioblastoma multiforme, joint segmentation, salvage radiotherapy

## Abstract

**Background:**

Salvage radiotherapy (SRT) with photons is a valid treatment option for patients suffering from recurrent glioblastoma (GBM). However, the tolerance of healthy brain to ionizing radiation (IR) is limited. The aim of this study was to determine to what extent brain structures in the radiographically tumor-free hemisphere change after repeated radiotherapy.

**Methods:**

Five of 26 patients treated with SRT for local recurrence of GBM were found to have magnetic resonance imaging (MRI) studies available for complete volumetric analysis before and after primary chemo-radiation and after SRT. Manual segmentation and joint segmentation (JS) based on a convolutional neural network were used for the segmentation of the gray matter, the white matter and the ventricles in T1 MRIs.

**Results:**

Qualitative results of manual segmentation and JS were comparable. After primary chemo-radiation and SRT, the volume of the contralateral ventricles increased steadily by 1.3–4.75% (SD ± 2.8 %, *R*^2^ = 0.82; *P* = <.01) with a manual segmentation and by 1.4–7.4% (SD 2.1%, *R*^2^ = 0.48; *P* = .025) with JS. The volume of the cortex decreased by 3.4–7.3% except in one patient, the cortex volume increased by 2.5% (SD ± 2.9%, *R*^2^ = 0.18; *P* = .19) when measured manually. When measured with JS GM decreased by 1.0–7.4%, in one case it increased by 3.0% (SD = 3.2%, *P* = .22, *R*^2^ = 0.18). The white matter remained stable when assessed with manual segmentation (*P* = .84, *R*^2^ = 0.004) or JS (*P* = .44, *R*^2^ = 0.07).

**Conclusion:**

SRT of relapsed GBM leads to continuous changes of the tumor-free contralateral brain by means of manual segmentation or JS. The cortex seems more susceptible to repeated RT compared to the white matter. Larger cohort studies and complementary functional analysis are encouraged.

**Key Points**

- Salvage radiotherapy of relapsed GBM does not accelerate the loss of brain tissue.
- Morphometry using convolutional neural networks is reliable after salvage radiotherapy.

**Importance of the Study**

This is the first study comparing manual and machine-based morphometry of the healthy brain after salvage radiotherapy suggesting that the loss of healthy brain tissue is neither enhanced nor accelerated after salvage radiotherapy.

Each year, around 7000 patients in Germany are newly diagnosed with a brain tumor, nearly 75% of those are classified as GBM (gliobastoma, World Health Organization Grade IV).^1^ The current standard of care for patients diagnosed with GBM includes maximum safe resection of the tumor, followed by fractionated radiotherapy and concomitant and adjuvant temozolomide.^2–4^ A significant prolongation of overall survival as well as progression-free survival by the addition of temozolomide to the therapy has been shown,^2,5^ and the combination with tumor treatment fields—low intensity alternating electric fields administered to the area of the tumor—to temozolomide therapy prolonged overall survival significantly as well, when compared with temozolomide-based chemoradiotherapy.^6^ More recently, Herrlinger et al. suggested a significant prolongation of overall survival combining temozolomide with lomustine compared with chemoradiation with temozolomide alone.^7^ Ninety percent of patients suffering from GBM need retreatment due to tumor relapsing.^8^ Currently, no standard of care for the treatment of recurrent GBM has been established.^8–11^ The most common options are re-resection, re-irradiation, or retreatment with temozolomide.^12^ Previous studies suggested that individual plans for each patient with recurrent GBM dependent on target volume, age, performance status, and response to and type of previous therapy and quality of life need to be established.^9,10^ The cumulative effect of repeated radiotherapy on healthy brain tissue has not been conclusively investigated. To observe changes in the tumor-free hemisphere of the brain after SRT is the main goal of this study.

Radiotherapy is a key modality in the treatment of GBM.^3^ With its development over the last 80 years, treatment has become more precise in targeting the tumor area, thus sparing nonaffected and otherwise healthy brain tissue.^3^ Despite technical improvement, radiotherapy is still associated with cognitive impairment and hemodynamic changes in the healthy brain tissue.^13,14^ Modern techniques have led to a sharp decline in the incidence of acute brain injury (days—weeks after radiotherapy) as well as early delayed brain injury (1 to 6 months after radiotherapy), late demyelination and white matter necrosis.^15^ Still, 60–90% of patients receiving brain radiation show symptoms of cognitive dysfunction for example in learning, memory, and spatial processing.^16–19^ Mechanisms contributing to the neurocognitive decline are thought to be damage in neural cells, endothelial cells, oligodendrocytes as well as the complex interaction between these different cell types, altered neurogenesis and increased neuroinflammation, most likely is a combination of the above.^15,16^

Structural changes and cortical thinning in the healthy hemisphere of the brain after radiotherapy have been found in recent studies. Although study design differs, they all suggest a certain toxicity of radiotherapy to the brain due to measured changes in brain structures. Decline of gray matter, expansion of ventricles and a loss of WM-integrity in the subventricular zone was found in follow-up MRIs in patients with GBM after receiving treatment consisting of systemic temozolomide and one series of cranial radiotherapy.^20^ Petr et al. also found significant decrease in gray and white matter in the healthy hemisphere in patients treated with photon-based radiotherapy. In this study, a dose-dependency of gray -matter-volume decrease was also found, while not finding any influence of chemotherapy duration on the volume decrease.^21^ In a review by Nagtegaal et al. in 2019, the authors collected results from a series of studies regarding the issue of changes in cortical thickness and volume after radiotherapy. The authors concluded that a connection between radiotherapy and thinning of the cortex exists, although the studies analyzed were too inhomogeneous to suggest any clinical changes of praxis.^22^

The effects of radiotherapy after tumor progression after primary multimodal therapy have not been investigated. This study aims to measure longitudinal effects of SRT on the volume of gray matter, white matter and the ventricles of the radiographically tumor-free hemisphere in patients diagnosed with relapsing GBM using manual and voxel-based morphometry.^23^

## Methods and Materials

### Patients

**Figure 1.**
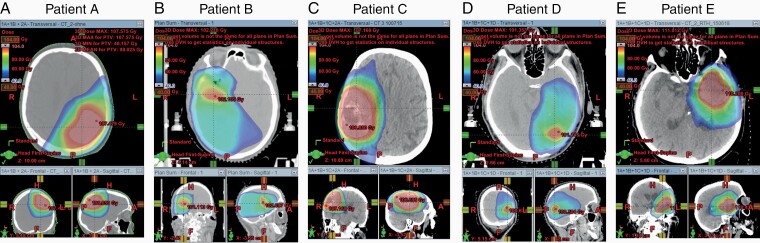
Sum-plans of primary and salvage conformal radiotherapy or intensity-modulated arc therapy. Red indicates areas treated with ≥100Gy.

Between 2009 and 2017, 26 patients underwent retreatment with photon radiotherapy for local recurrence of a GBM at the department of radiotherapy, City Hospital Dessau, Dessau, Germany. All patients’ consent for data analysis regarding quality and outcome assessment was available prior to analysis. The ethical committee of the medical board of Saxony-Anhalt and the internal review Board of the Städtische Klinikum Dessau, Germany, granted the rights to publish clinical outcome analysis after salvage radiation therapy and the present case series. Briefly, their treatment consisted of the following: radical resection and a primary course of adjuvant chemo-radiation with 6 MV photons from a linear accelerator with a daily dose of 1.8–2.0 Gy given to a total dose of 59.4–63 Gy and concomitant chemotherapy with 75 mg/m^2^ temozolomid were followed by adjuvant temozolomid therapy at a dose of 200 mg/m^2^ for 6 months or until disease recurrence. Salvage radiotherapy was given using a daily fraction of 1.6–3 Gy treated to a total dose of 39–54 Gy as reported previously.^24^ Patients with unilateral disease and complete MRI studies before primary radiotherapy as well as before and after SRT (salvage radiotherapy) were eligible. Complete data for analysis were available for 5 patients, 2 female and 3 male patients with an average age of 61 years (range 46–76). Patient characteristics are specified in Table 1. Treatment plans of the eligible patients are illustrated in Figure 1a–e. The MRI after first radical resection prior to chemoradiation was taken as baseline data defining t_0_. The last MRI prior to SRT defined timepoint t_1._ MRIs available after SRT defined t_2._ The time to MRI available for the first biometric analysis (t_1_) was 245,4 (SD 24,1) days and 546,2 (SD 80,1) days for biometric analysis at (t_2_).

**Table 1.** Patient Characteristics

Patient	A	B	C	D	E
Gender	F	m	m	m	f
Age at diagnosis of glioblastoma	71	57	45	60	75
Died at age	72	59	47	Alive with 76 years	Alive with 62 years
Side affected by tumor	Left	Right	Right	Left	Left
Surgery to start SRT in days	462	419	486	278	323
Dose RT1 (Gy)	60	60	60.4	60	60
Dose SRT (Gy)	45	54	41	39	45
Salvage-PTV (mL)	68.23	94.8	138.02	5.11	23.49

f, female; m, male; PTV, planning target volume; RT, radiotherapy; SRT, salvage radiotherapy.

### Data

T1 MRI images were used to analyze the volume of the gray matter, the white matter, and the ventricles of the healthy hemisphere of the brain. MRI data were heterogeneous derived from outpatient clinics in Saxony-Anhalt, Germany, using 1.5 T or 3.0 T imaging devices. Only the MRI at t_0_ was uniformly obtained with a 3.0 T (Philips-Achieva, Hamburg, Germany).

### Data Processing

MRI studies were anonymized as the original DICOM files were transformed into Nifty-Images (.nfti) and all personal data stored in the original images deleted (ITK SNAP, version 3.5, Philadelpiha, PN^25,26^). The radiographically tumor-free hemisphere of the brain was segmented manually using ITK Snap (Version 3.5) to visualize and define the volume of the gray matter (GM), the white matter (WM), and the supratentorial ventricle (V).^25,26^ Volumes were assessed in mL and then normalized to half of the volume of the supratentorial cavity of the skull. The volume of the supratentorial cavity of the skull was assessed by betsurf by BET (brain extraction tool)^27^ and corrected manually with ITK SNAP (Version 3.5).^25,26^ Initial data were used for normalization to 100%, and further assessment of the change in volume was carried out in percentages. To control for manual segmentation, automated segmentation was performed with JS. The software (SW) required the skull of the MRI to be stripped of any bone. This was achieved using BET.^27^ Segmentation by JS was done as described previously (Himmetoglu M, Chen X, Konukoglu E, unpublished data). The JS model used a partially labeled dataset basing on the hypothesis that healthy tissues in different task-specific datasets follow a similar distribution, and the consistency for these latent variables can be used as a regularizer in the training of the network. A model based on Variational Autoencoder (VAE) was trained and assumed in the bottleneck between the encoder and decoder a 3-way clustered latent space: lesion image, healthy parts along in a brain image with lesion, and healthy brain images. Training in a multitask learning fashion enabled the model to learn to unify partially labeled and fully labeled datasets’ target space (Himmetoglu M, unpublished data). Results created in this way were then corrected manually to analyze only the tumor-free hemisphere of the brain. The SW worked with the same T1 images that were used for manual segmentation. The third timepoint (t_2_) of patient A was not available for joint segmentation due to lack of a sufficient MRI. The preprocessing of the imaging data run through JS was improved for a second analysis by adjusting the contrast and matching the histograms of test and training data. Segmentation done with improved preprocessing did not need manual correction, because the SW was able to perform the segmentation on the healthy side of the hemisphere only, in contrast to the first analysis, where the tumor-affected hemisphere had to be removed from segmentation manually. Segmentation performed with improved preprocessing is labeled as joint segmentation 2 (JS2).

### Statistical Analysis

Longitudinal changes as a function of time (days) were assessed using linear regression. A *P*-value of < .05 was considered statistically significant. To carry out the statistical analysis, the combined volume of the gray matter, the white matter, and the ventricles were normalized to 100% of the healthy half of the supratentorial brain cavity. All statistical analyses were performed on Excel (Version 16.16.25).

## Results

**Figure 2.**
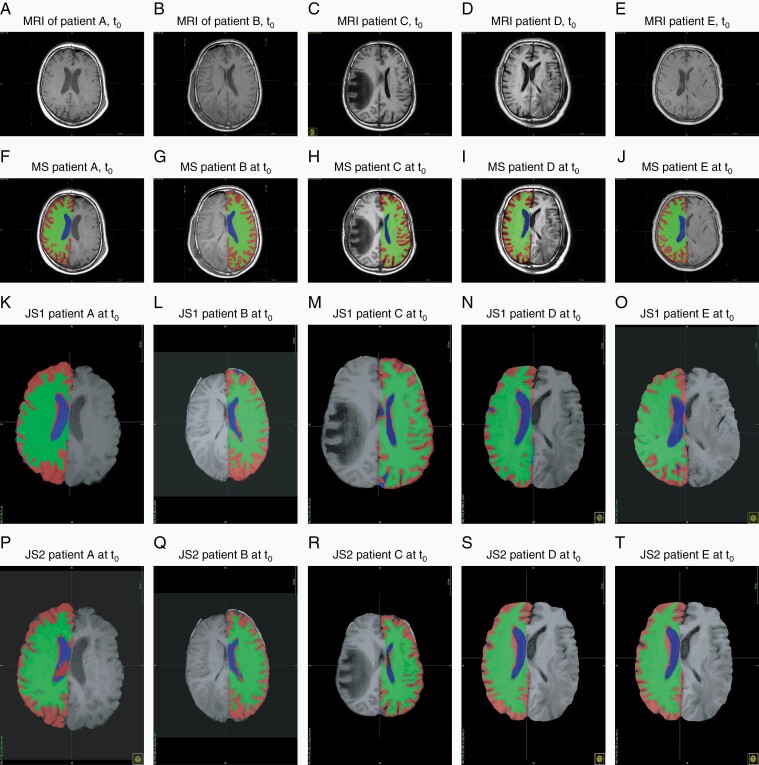
Manual segmentation and deep learning based joint segmentation. T1 MRI prior to radiotherapy (2a–e), with manual segmentation (MS, 2f–j), joint segmentation 1 (JS1, 2k–o), and joint segmentation 2 (JS2, 2p–t). White matter is in green, gray matter in red, and ventricles in blue.

Imaging data after gross tumor resection, 6 months after the end of the first radiotherapy-cycle and 2 to 6 months after salvage radiotherapy were available for 5 out of 26 patients only (19%) (Figure 2a–e). Figure 2 illustrates the difference in the quality of segmentation between manual segmentation (Figure 2f–j) and segmentation performed by a deep learning program (Figure 2k–t) for the same segment of the image of patients A to E at t_0_. In Figure 2p–t, the segmentation was performed by the deep learning program after improved preprocessing. We expected more precise segmentation in Figure 2p–t than in Figure 2k–o, which was not the case.

Looking at the segmented images, manual segmentation appears to be more detailed, especially at the segmentation of the gyri. Manual segmentation gave volumes smaller than the JS. The volume of the GM was 9.87 % bigger when measured with JS (SD = 21.47%) and WM volumes were 1.08% larger when assessed by JS (SD = 10.26%). The volumes of the ventricles assessed by JS and manual segmentation had the biggest discrepancy with JS volumes being 65.10% bigger than manually assessed volumes of the ventricles (SD = 48.89%).

**Figure 3.**
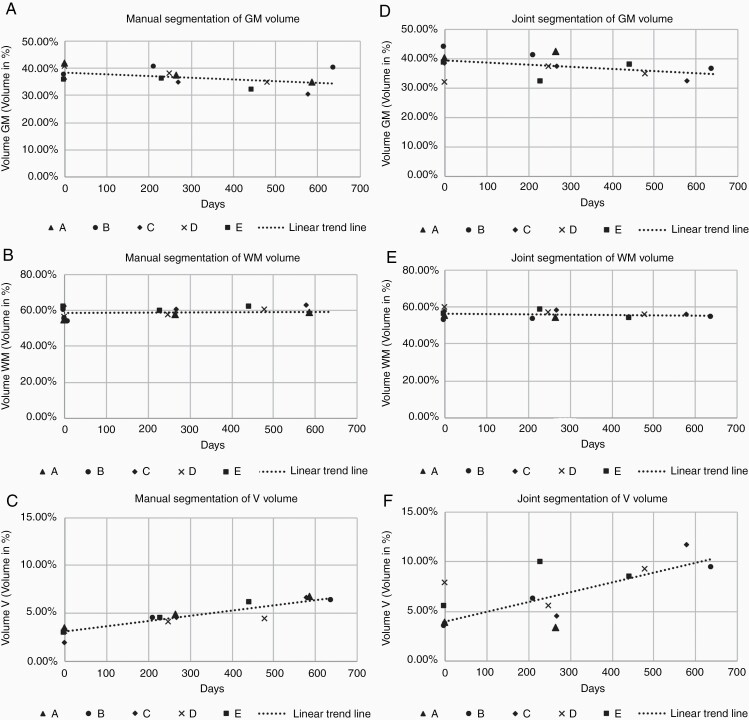
Changes of brain volume in the gray matter (a,d) white matter (b,e), and the ventricles (c,f) as a function of time.

The changes of brain substance over time are illustrated in Figure 3 after being assessed manually by voxel morphometry (Figure 3a–c) and by JS (Figure 3d–f). 100% equals the combined volume of GM, WM, and V.

### Gray Matter

The gray matter decreased in 4 cases by 3.4–7.3% over the analyzed time period. In one case gray matter volume increased by 2.5% (SD = 2.9%) from t_0_ to t_2_. Statistical regression analysis of the change in GM showed no significant change of the volume of GM (*P* = .19, *R*^2^ = 0.18). Findings were confirmed with JS with following results: The gray matter decreased in 3 cases by 1.0–7.4%, in one case the volume increased by 3.0% respectively (SD = 3.2%) from t_0_ to t_2_. Statistical regression analysis showed no significant change (*P* = .22, *R*^2^ = 0.18).

### White Matter

The volume of the white matter remained stable during the observed time period by means of manual segmentation. The change ranges from an increase of 4.5% to a decrease of 5.9% (SD = 2.8%). Regression analysis showed no significant change of the volume of WM (*P* = .84, *R*^2^ = 0.004). Similarly, no significant changes by JS were found in the analyzed time period (*P* = .44, *R*^2^ = 0.07).

### The Ventricles

The volume of the ventricles increased in a range from 1.3% to 4.75% (SD = 0.5%) with manual segmentation. Compared to their original volumes, this is an increase by up to 200% (SD = 53%). The regression analysis showed a significant change of the volume of the ventricles (*P* < .01, *R*^2^ = 0.82). For JS, the volume of the ventricles increased by 1.4%–7.4%. The increase was significant (*P* = .025, *R*^2^ = 0.48).

To better illustrate the obtained results, measurements from patient C are shown in more detail. Given that for our study, the combined volume of the analyzed gray matter, white matter and ventricles of the hemisphere not affected by tumor-mass was defined as 100%, gray matter volume at t_0_ was 36.03% when measured manually and 38.29% when measured with JS. The measured values for white matter at t_0_ were 62.04% (MS) and 57.2% (JS). The ventricles at t_0_ were 1.91% (MS) and 3.90% (JS). The patient then underwent treatment with temozolomide and primary radiotherapy.

The MRI at t_1_ was acquired 269 days after t_0_ and showed gray matter volumes of 34.84 % (MS) and 37.25% (JS). White matte volume at t_1_ was 60.57% (MS) and 58.24% (JS), ventricles were measured to have a volume of 4.59 % (MS) and 4.51% (JS) at t_1_. Gray and white matter decreased in volume between t_0_ and t_1_ when measured with manual segmentation and also when measured with joint segmentation. Ventricles increased in volume between t_0_ and t_1._

GBM relapsed and patient C underwent SRT. After the end of re-radiation, the MRI at t_2_ was taken, 578 days after t_0_. Gray matter volume at t_2_ was 30.51% (MS) and 32.31% (JS), white matter volume at t_2_ was 62.83% (MS) and 56.05% (JS), the volume of the ventricle at t_2_ was 6.66% (MS) and 11.64% (JS). This shows a further decrease in gray matter volume compared to t_0_, a stable volume measured for white matter between t_0_ and t_2_ and an increase of the ventricle volume in the timespan from t_0_ and t_2_.

## Discussion

The preservation of healthy and functional brain tissue is a major challenge for treating brain tumors, and in case of relapsing brain tumors, efforts of tumor control must be well balanced against additional toxicity. The impact of chemoradiation on brain structures after treatment of brain tumors has been well recognized.^20–22,28^ However, in how far additional radiotherapy to the brain may enhance damage has not been elucidated. Here we show that SRT does not seem to accelerate the loss of brain structures after primary chemoradiation because structural changes appear to be linear over time. This finding is important because enhanced tissue damage might be expected due to the limited ability of brain tissue to compensate for cumulative high doses of radiation exceeding 100 Gy. Thus, our pilot study highlights the utility and value of focal radiotherapy for relapsing tumor disease. SRT likely merits be listed up in popular guidelines on relapsing GBM, such as those provided by the NCCN.^4^

Our observations regarding brain tissue after SRT are largely in agreement with the observations by Prust et al.^20^ and Petr et al.,^21^ despite some differences in patient selection, data processing, and findings. Both research groups included patients with unilateral tumors and observed volume changes in the contralateral tumor-free hemisphere after primary therapy. Prust et al. compared imaging data taken before RCT and then weekly during RCT and monthly after RCT up to 6 months, Petr et al. used MRI from before RCT, 3 and 6 months after the end of RCT. Differences in the loss of GM that has been observed in the present study and the even more impressive decrease in GM observed in previous observations, can be mostly explained by sample size and more standardized MRI acquisition in previous protocols.^20,21^

Automated segmentation of patients MRI generates results free from intra-observer bias. Automated segmentation has the advantage of being much faster than manual segmentation, which enables researchers to analyze bigger patient samples and therefore obtain more conclusive results. Due to the severity of the disease and the clinical prognosis, however, continuous imaging documentation is challenging. Heterogenous studies are difficult to be analyzed by software segmentation because often no standard protocol has been followed. Automated segmentation by JS is able to provide reliable results given the MRIs have MRI protocol following Table 2. The SW is also capable of segmenting the tumor, for which FLAIR sequences are necessary. Additionally, T2 sequences are needed in order to have detailed enough skull stripping by BET.^27^ Despite good results given by joint segmentation, manual segmentation of gray matter, white matter and the ventricles are still considered the gold standard in segmentation as intra-observer variability is less than 10% (data not shown), although manual segmentation is time consuming and not applicable for clinical routine.

**Table 2.** MRI Sequences and Parameters

Sequence	TR/TE (ms)	FA	ST (mm)	FOV (cm)	Matrix
T1-weighted	459/10	90°	5	23	512 × 512

FA, flip angle; FOV, field of view; ST, slice thickness; TE, echo time; TR, repetition time.

Overall, JS and manual segmentation gave comparable results. Segmentation of the ventricles was easily achieved manually, and the JS-based volumes assessment was 65.10% larger than the manual volumes. The difference is explained by the fact, that JS scored the cerebrospinal fluid around the brain as well. In contrast, segmentation of the GM and WM with JS was more consistent with manual segmentation. The differences of manual and machine-based segmentation with JS was around 10 % for GM (cf. Results), and around 1% for the WM. The differences of accuracy are likely reflecting segmentation insecurity in the context of the peri-cerebral fluids.

JS measurements had bigger mean variations than manual segmentation, likely caused by the lack of detailed segmentation by JS. Another problem experienced with JS was the different intensity of the same compartment in different MRIs because the JS is not only based on the programs experience on where to find different compartments but also on hard cut-offs like the intensity of a voxel. The problem occurred when segmenting the ventricles as the intensity of the cerebrospinal fluid varied between MRIs. This caused the JS to allocate parts of the ventricles to other compartments as seen in Patient E, which explains the low ventricle volume found by JS at the timepoint after salvage radiation.

Results obtained in this study show changes in healthy brain tissue in the tumor-free hemisphere in patients with GBM that have been treated with repeated radiotherapy and concomitant and adjuvant temozolomide.

Decrease of gray matter after primary radiotherapy in patients with GBM has been shown in several studies.^20,22,28^ In our study, the decrease of GM has not been significant, despite similar definition of healthy brain and comparable measurement methods. The most obvious explanation is the number of analyzed patients. While we only had 5 patients with sufficient MRI available for analysis, Prust et al. observed 14 patients, Petr et al. analyzed 51 patients treated after primary photon radiotherapy. Limited sample size causes effects to go undetected and to lack significance.^29^ Studies with bigger sample sizes examining changes in brain volume after SRT are needed to retrieve more conclusive results. Most important of all, the decline of GM, even though not statistically significant, was linear and not accelerated.

The increase of ventricles mirrored the decrease of the volumes of the GM. Ventricles increased linearly over time after the first radiotherapy and SRT. This finding is especially interesting in the light of previous observations that showed a more distinctive neurocognitive decline in patients with tumor progression.^30^ In contrast to these earlier findings,^30^ continuous decrease of GM and increase of the ventricles were observed after tumor progression and SRT in our patients. In a previous study, an increase of the ventricles and loss in GM was observed to be correlated, which could indicate that changes in ventricular volume can serve as a surrogate for cortical atrophy.^20^ In patients with dementia, the connection between increase of the ventricle volume and decrease of GM has been shown,^31^ so this correlation could be of clinical significance, when an increase of ventricles and a decrease of the GM eventually correlate with neurocognitive impairment.

The decrease of the volume of the cortex has been known to be a physiological feature of aging.^32,33^ The decrease of the GM volume has been reported to be ~1.6%/decade.^32^ In our patients, a decrease of 11.3% (SD ± 19 %) in 2 years was noticed, exceeding the physiological regression of GM by aging by far.

The volume of the ventricles increases physiologically during aging by ~10.9% per decade.^32^ In the present series, the ventricles in the healthy hemisphere of patients, treated with 2 courses of radiotherapy increased by 102% (SD ± 61.57%) over 2 years.

The effect of radiotherapy on the volume of the WM has been less well documented in the literature. Chemoradiotherapy was reported to lead to changes in WM.^34^ However, in the present small series, we observed no morphometric changes over the time of 546 days (SD ± 72). Prust et al., who observed similar results in regard to the nonsignificant change in WM, discussed that the change from whole brain radiotherapy to focal therapy and thus less irradiated brain-mass leads to the fewer changes in the volume of the white matter.^20^ However, in a subsequent paper published 2 years later by the same authors, significant WM loss was reported.^28^ This inconsistency was explained by the larger patient sample size analyzed in the second report and the more extended follow-up time and additional therapy with cediranib, a vascular endothelial growth factor- inhibitor, after radio-chemotherapy.^28^

Some limitations of this study need to be considered. Obviously, the sample size with only 5 patients with available T1 imaging data for all required time points was small. Limited sample size causes effects to go undetected and to miss significance.^29^ The fact, that in our cohort only 20% of the patients had MRIs 6 months after SRT can be explained by reluctance to repeat imaging studies in the context of palliative patient management after GBM recurrences. Cohort studies with bigger patient samples are likely to result in more compelling conclusions. Still, our results are in line with observations of brain volume change after primary radio-chemotherapy. Furthermore, by the retrospective design, controlling for sex, age nor other possibly influential factors of brain-volume was not possible, and no control group was available. Another methodological weakness is the fact that the last imaging series analyzed varied in timespan from the end of the SRT, as images 6 months after the end of SRT were available for a minority of patients only. Images analyzed at different time points could show different stages of radiotherapy-induced atrophy and maybe reversal of radiotherapy-induced damage, as well. Another shortcoming of our study is the lack of neurocognitive testing. Functional endpoints will be important to obtain in a second step.^35,36^ An extensive review conducted by Lawrie et al. in 2019 showed that radiotherapy for glioma may increase the risk for cognitive impairment and neurocognitive testing should be performed as part of long-term follow-up.^14^ Life expectations of patients with GBM, as observed in this study, are often limited, which makes long-term follow-up difficult.

## Conclusion

Our results reveal continuous changes over time of the healthy contralateral brain after repeated irradiation of GBM. Most pronounced changes are observed to be linear, mainly of the ventricles and to a lesser degree of the gray substance. Segmentation with AI and deep learning is comparable with manual segmentation although manual segmentation remains the gold standard due to better discrimination and resolution of the structural compartments. The present findings define the need to further assess long-term impact on brain structures and compartments after SRT and ask for correlation with functional analysis.

## Notes

**Authorship Statement**. L.dlC. reviewed all patients and performed the manual segmentation, analyzed the data from manual and machine-based segmentation; she wrote the manuscript. X.C. was responsible for joint segmentation and machine-based segmentation of the set and was involved in writing of the manuscript. E.K. supervised the process of machine-learned segmentation and provided the computer facilities, was responsible for data interpretation and approved the final version of the manuscript. I.F.C.: treated all patients, planned and designed the study, supervised segmentation, was responsible for interpretation and publication of study.

## References

[1] Kaatsch P , Spix C, Katalinic A, et al Krebs in Deutschland für 2015/ 2016. Berlin: Robert Koch-Institut (Hrsg) und die Gesellschaft der epidemiologischen Krebsregister in Deutschland e.V. (Hrsg); 2019.

[2] Stupp R , Mason WP, van den Bent MJ, et al.; European Organisation for Research and Treatment of Cancer Brain Tumor and Radiotherapy Groups; National Cancer Institute of Canada Clinical Trials Group. Radiotherapy plus concomitant and adjuvant temozolomide for glioblastoma. N Engl J Med. 2005;352(10):987–996. 15758009 10.1056/NEJMoa043330

[3] Gzell C , Back M, Wheeler H, Bailey D, Foote M. Radiotherapy in Glioblastoma: the Past, the Present and the Future. Clin Oncol (R coll radiol). 2017;29(1):15–25. 27743773 10.1016/j.clon.2016.09.015

[4] Nabors LB , Portnow J, Ahluwalia M, et al Central nervous system cancers, version 3.2020. JNCCN Journal of the National Comprehensive Cancer Network. 2020;18(11):1537–70. 33152694 10.6004/jnccn.2020.0052

[5] Perry JR , Laperriere N, O’Callaghan CJ, et al Short-course radiation plus temozolomide in elderly patients with glioblastoma. N Engl J Med. 2017;376(11):1027–37. doi: 10.1056/NEJMoa1611977. 28296618

[6] Stupp R , Taillibert S, Kanner A, et al. Effect of tumor-treating fields plus maintenance Temozolomide vs Maintenance Temozolomide alone on survival in patients with Glioblastoma: a randomized clinical trial. JAMA. 2017;318(23):2306–2316. 29260225 10.1001/jama.2017.18718 PMC5820703

[7] Herrlinger U , Tzaridis T, Mack F, et al.; Neurooncology Working Group of the German Cancer Society. Lomustine-temozolomide combination therapy versus standard temozolomide therapy in patients with newly diagnosed glioblastoma with methylated MGMT promoter (CeTeG/NOA-09): a randomised, open-label, phase 3 trial. Lancet. 2019;393(10172):678–688. 30782343 10.1016/S0140-6736(18)31791-4

[8] Weller M , Cloughesy T, Perry JR, Wick W. Standards of care for treatment of recurrent glioblastoma–are we there yet? Neuro Oncol. 2013;15(1):4–27. 23136223 10.1093/neuonc/nos273 PMC3534423

[9] Scoccianti S , Francolini G, Carta GA, et al. Re-irradiation as salvage treatment in recurrent glioblastoma: a comprehensive literature review to provide practical answers to frequently asked questions. Crit Rev Oncol Hematol. 2018;126:80–91. 29759570 10.1016/j.critrevonc.2018.03.024

[10] Seystahl K , Wick W, Weller M. Therapeutic options in recurrent glioblastoma–An update. Crit Rev Oncol Hematol. 2016;99:389–408. 26830009 10.1016/j.critrevonc.2016.01.018

[11] Stupp R , Wong ET, Kanner AA, et al. NovoTTF-100A versus physician’s choice chemotherapy in recurrent glioblastoma: a randomised phase III trial of a novel treatment modality. Eur J Cancer. 2012;48(14):2192–2202. 22608262 10.1016/j.ejca.2012.04.011

[12] Nam JY , de Groot JF. Treatment of glioblastoma. J Oncol Pract. 2017;13(10):629–638. 29020535 10.1200/JOP.2017.025536

[13] Kłos J , van Laar PJ, Sinnige PF, et al. Quantifying effects of radiotherapy-induced microvascular injury; review of established and emerging brain MRI techniques. Radiother Oncol. 2019;140:41–53. 31176207 10.1016/j.radonc.2019.05.020

[14] Lawrie TA , Gillespie D, Dowswell T, et al. Long-term neurocognitive and other side effects of radiotherapy, with or without chemotherapy, for glioma. Cochrane Database Syst Rev. 2019;8:CD013047. 31425631 10.1002/14651858.CD013047.pub2 PMC6699681

[15] Greene-Schloesser D , Robbins ME, Peiffer AM, Shaw EG, Wheeler KT, Chan MD. Radiation-induced brain injury: a review. Front Oncol. 2012;2:73. 22833841 10.3389/fonc.2012.00073 PMC3400082

[16] Makale MT , McDonald CR, Hattangadi-Gluth JA, Kesari S. Mechanisms of radiotherapy-associated cognitive disability in patients with brain tumours. Nat Rev Neurol. 2017;13(1):52–64. 27982041 10.1038/nrneurol.2016.185 PMC5805381

[17] Pazzaglia S , Briganti G, Mancuso M, Saran A. Neurocognitive decline following radiotherapy: mechanisms and therapeutic implications. Cancers (Basel). 2020;12(1):146. 10.3390/cancers12010146 PMC7017115 31936195

[18] Hilverda K , Bosma I, Heimans JJ, et al. Cognitive functioning in glioblastoma patients during radiotherapy and temozolomide treatment: initial findings. J Neurooncol. 2010;97(1):89–94. 19718545 10.1007/s11060-009-9993-2 PMC2814037

[19] Hottinger AF , Yoon H, DeAngelis LM, Abrey LE. Neurological outcome of long-term glioblastoma survivors. J Neurooncol. 2009;95(3):301–305. 19557499 10.1007/s11060-009-9946-9

[20] Prust MJ , Jafari-Khouzani K, Kalpathy-Cramer J, et al. Standard chemoradiation for glioblastoma results in progressive brain volume loss. Neurology. 2015;85(8):683–691. 26208964 10.1212/WNL.0000000000001861 PMC4553035

[21] Petr J , Platzek I, Hofheinz F, et al. Photon vs. proton radiochemotherapy: Effects on brain tissue volume and perfusion. Radiother Oncol. 2018;128(1):121–127. 29370984 10.1016/j.radonc.2017.11.033

[22] Nagtegaal SHJ , David S, van der Boog ATJ, Leemans A, Verhoeff JJC. Changes in cortical thickness and volume after cranial radiation treatment: a systematic review. Radiother Oncol. 2019;135:33–42. 31015168 10.1016/j.radonc.2019.02.013

[23] Ashburner J , Friston KJ. Voxel-based morphometry–the methods. Neuroimage. 2000;11(6 Pt 1):805–821. 10860804 10.1006/nimg.2000.0582

[24] Ciernik IF , Gager Y, Renner C, Spieker S, Arndt N, Neumann K. Salvage radiation therapy for patients with relapsing glioblastoma multiforme and the role of slow fractionation. Front Oncol. 2020;10:577443. 33364191 10.3389/fonc.2020.577443 PMC7753368

[25] Yushkevich PA , Yang G, Gerig G. ITK-SNAP: an interactive tool for semi-automatic segmentation of multi-modality biomedical images. Annu Int Conf IEEE Eng Med Biol Soc. 2016;2016:3342–3345. 28269019 10.1109/EMBC.2016.7591443 PMC5493443

[26] Yushkevich PA , Piven J, Hazlett HC, et al. User-guided 3D active contour segmentation of anatomical structures: significantly improved efficiency and reliability. Neuroimage. 2006;31(3):1116–1128. 16545965 10.1016/j.neuroimage.2006.01.015

[27] Smith SM . Fast robust automated brain extraction. Hum Brain Mapp. 2002;17(3):143–155. 12391568 10.1002/hbm.10062 PMC6871816

[28] Prust ML , Jafari-Khouzani K, Kalpathy-Cramer J, et al. Standard chemoradiation in combination with VEGF targeted therapy for glioblastoma results in progressive gray and white matter volume loss. Neuro Oncol. 2018;20(2):289–291. 29315410 10.1093/neuonc/nox217 PMC5777497

[29] Noordzij M , Dekker FW, Zoccali C, Jager KJ. Sample size calculations. Nephron Clin Pract. 2011;118(4):c319–c323. 21293154 10.1159/000322830

[30] Bosma I , Vos MJ, Heimans JJ, et al. The course of neurocognitive functioning in high-grade glioma patients. Neuro Oncol. 2007;9(1):53–62. 17018697 10.1215/15228517-2006-012 PMC1828106

[31] Madsen S , Gutman B, Joshi S, et al Mapping ventricular expansion onto cortical gray matter in older adults. Neurobiol Aging. 2015;36(suppl. 1):32–41. 10.1016/j.neurobiolaging.2014.03.044 PMC4268107 25311280

[32] Blatter DD , Bigler ED, Gale SD, et al. Quantitative volumetric analysis of brain MR: normative database spanning 5 decades of life. Am J Neuroradiol. 1995;16(2):241–251. 7726068 PMC8338340

[33] Salat DH , Buckner RL, Snyder AZ, et al. Thinning of the cerebral cortex in aging. Cereb Cortex. 2004;14(7):721–730. 15054051 10.1093/cercor/bhh032

[34] Simó M , Rifà-Ros X, Rodriguez-Fornells A, Bruna J. Chemobrain: a systematic review of structural and functional neuroimaging studies. Neurosci Biobehav Rev. 2013;37(8):1311–1321. 23660455 10.1016/j.neubiorev.2013.04.015

[35] Simó M , Vaquero L, Ripollés P, et al. Longitudinal brain changes associated with prophylactic cranial irradiation in lung cancer. J Thorac Oncol. 2016;11(4):475–486. 26804637 10.1016/j.jtho.2015.12.110

[36] Bergo E , Lombardi G, Guglieri I, Capovilla E, Pambuku A, Zagone V. Neurocognitive functions and health-related quality of life in glioblastoma patients: a concise review of the literature. Eur J Cancer Care (Engl). 2019;28(1):e12410. 26531122 10.1111/ecc.12410

